# Contradictions in the promotion of publishing academic and scientific journal articles, and the inability to cope with the new coronavirus (COVID-19)

**DOI:** 10.1186/s13756-021-00884-0

**Published:** 2021-01-12

**Authors:** Rostam Jalali, Amin Hosseinian-Far, Masoud Mohammadi

**Affiliations:** 1 Department of Nursing, School of Nursing and Midwifery, Kermanshah University of Medical Sciences, Kermanshah, Iran; 2 Department of Business Systems and Operations, University of Northampton, Northampton, UK

**Keywords:** COVID-19, Publishing, Journal article, Research ethics, Research policy

## Abstract

**Background:**

Translating research into practice is a central priority within the National Institutes of Health (NIH) Roadmap. The underlying aim of the NIH Roadmap is to accelerate the movement of scientific findings into practical health care provisions through translational research.

**Main text:**

Despite the advances in health sciences, emerging infectious diseases have become more frequent in recent decades. Furthermore, emerging and reemerging pathogens have led to several global public health challenges. A question, and to an extent a concern, arises from this: Why our health care system is experiencing several challenges in encountering the coronavirus outbreak, despite the ever-growing advances in sciences, and the exponential rise in the number of published articles in the first quartile journals and even the ones among the top 1%?

**Conclusion:**

Two responses could be potentially provided to the above question: First, there seems to be a significant gap between our theoretical knowledge and practice. And second that many scholars and scientists publish papers only to have a longer list of publications, and therefore publishing is viewed as a personal objective, rather than for improving communities’ public health.

## Background

Translational research is an evolving concept. As defined by NIH, Type 1 Translation applies basic scientific discoveries to human health care under controlled conditions. Type 2 Translation promotes the adoption of the outcomes of promising clinical research by community-based health care under uncontrolled and often uncontrollable conditions [1]. Translating research into practice is a central priority within the National Institutes of Health Roadmap. The underlying aim of the NIH Roadmap is to accelerate the movement of scientific findings into practical health care provision through translational research, so that the improvements in the health of the nation can be realized [2].

Yet, has the society really progressed with the application of knowledge at the same pace of the rise in the number of published scientific articles, the increase of society's knowledge about diseases, prevention mechanisms, screening. and treatment approaches, and the improvement of general public health?

Today, the entire world is challenged by the Covid-19 pandemic. Consequently, this imperative question arises: Why our health care system is experiencing several challenges in encountering the coronavirus outbreak, despite the ever-growing advances in sciences, and the exponential rise in the number of published articles in the first quartile (Q1) journals and even the ones among the top 1%?

## Main text

Providing a response to the above question requires a great deal of thought and foresight; a number of sub questions emerge as a result: What is the main purpose of publishing articles? Is the main aim of published articles really to raise public awareness and to promote critical thinking within scientific communities to combat diseases?

All academic-scholarly journals have their own stated objectives, agenda and scope, i.e., the particular research topics or themes, and research types that each journal covers. Having detailed information about the most prolific authors, most-frequently explored topics, single, co-, or multiple authorship, international contributions, various academic institutions' scholarly contributions, and the graduate or doctoral students' contributions can be of interest and significance to it’s the immediate stakeholders (its publisher, editors, and editorial board), and can also potentially impact health care systems [3].

On the other hand, although it seems paradoxical, considering the advances in health and medical sciences, emerging infectious diseases (EIDs) have become more frequent in recent decades^4^. Emerging and reemerging pathogens are among the key global challenges for public health systems. This seems even more paradoxical with respect the scientific effort to combat the new coronavirus. Coronaviruses are enveloped RNA viruses that are distributed broadly among humans, other mammals, and birds, and can cause respiratory, enteric, hepatic, and neurologic diseases. The initial cases of the novel coronavirus (2019-nCoV)–infected pneumonia (NCIP) were reported in Wuhan, Hubei Province, China, in December 2019 [4].

The Virus spread rapidly in all continents, and the international, national, and regional statistical reporting on the number of infections, and mortalities have become part of the day-to-day life in several countries. Simultaneously, biomedical and pharmaceutical companies have started the race to find a low-risk treatment or a trustworthy vaccine. Moreover, the scientific community have engaged extensively in scientific research projects, and consequently the number of COVID-19 related research articles is rising swiftly. In Iran, for instance, according to statistics reported by the World Health Organization (WHO), on August 27, 2020, the number of reported cases and deaths due to Covid-19 were 363,363 and 20,901, respectively [5], while only in the Scopus database [6], Iranian authors have registered 26,709 articles in 2020 alone. Scopus is the largest abstract and citation database of peer-reviewed literature, with bibliometrics tools to track, analyze and visualize research. It contains over 22,000 titles from more than 5,000 publishers around the world, covering the fields of science, technology, medicine, social sciences, and arts & humanities. Scopus has 55 million records dating back to 1823, 84% of these contain references dating from 1996 [6].

Before this particular pandemic, at least two similar epidemics were experienced in the world, i.e. the H1N1 and H5N1 influenza epidemics. Thus, several countries have had the experience of, rather successfully, dealing with epidemics, yet with the COVID-19, the disease has spread globally with high mortality rates, resulting in financial, social and political crises in several countries, with potentially many other upcoming consequences, many of which are currently unknown. Washing hands with soap and water, covering the nose and mouth when sneezing, social distancing, as well as avoiding close contact with people with the disease symptoms are listed among the most important preventive measures by WHO. Despite the simplicity of these measures, the pandemic is still spreading.

Nonetheless, there does not seem to be any shortcomings in regard to the engagement of scientific communities. In fact, the number of COVID-19 related research articles, published in the first quartile (Q1) journals, has been rising exorbitantly. This demonstrates that there is a gap between increasing and promoting science and the rise in the number of published articles, and their effectiveness within societies.

Topics that affect the entire world, such as epidemics and pandemics, are increasingly being considered by researchers, most notably the COVID 19 pandemic, with the greatest focus on the nature of the virus, the symptoms of patients, how they are diagnosed and presented. It is a diagnostic and therapeutic solution that shows the main essence of research, which is to solve problems and answer questions.

But in practice we only see articles that are widely published and actually increase the number of articles and do not help to solve the problem, the report of the number of published articles speaks for itself, although our thinking is that It is that as the number of articles increases, so does the public awareness and the health status of the people, but in practice, such a thing has a wide gap between the number of articles published and the awareness of the people and the community.

## Conclusion

A response to the highlighted concern can be provided from two perspectives: First, there seems to be a significant gap between theoretical knowledge and practice. And second that the many scholars and scientists publish papers only to have a longer list of publications. Therefore, it seems that in many cases, publishing is a personal objective, rather than for improving communities’ public health. These personal goals can be a slight increase in the number of articles, an increase in the number of citations and an increase in the H-index, and the researcher is known as a prominent researcher in the community, while practically no effort has been made to improve community health. Finding a response to the questions raised within the letter may help us to redirect our research and publishing strategy. Moreover, such responses may inform related policies to be devised by relevant stakeholders, and in particular, the Ministry of Health and Medical Education.

## Data Availability

Not applicable.
